# Topotactic Growth of Zintl Phase Eu_5_In_2_As_6_ Nanowires with Antiferromagnetic Behavior

**DOI:** 10.1021/acs.nanolett.5c00008

**Published:** 2025-03-11

**Authors:** Man Suk Song, Lothar Houben, Nadav Rothem, Ambikesh Gupta, Shai Rabkin, Beena Kalisky, Haim Beidenkopf, Hadas Shtrikman

**Affiliations:** † Department of Condensed Matter Physics, Weizmann Institute of Science, Rehovot 7610001, Israel; ‡ Department of Chemical Research Support, Weizmann Institute of Science, Rehovot 7610001, Israel; § Department of Physics and Institute of Nanotechnology and Advanced Materials, Bar-Ilan University, Ramat Gan 5290002, Israel

**Keywords:** MBE, nanowires, Zintl phase, topotaxy, antiferromagnetism

## Abstract

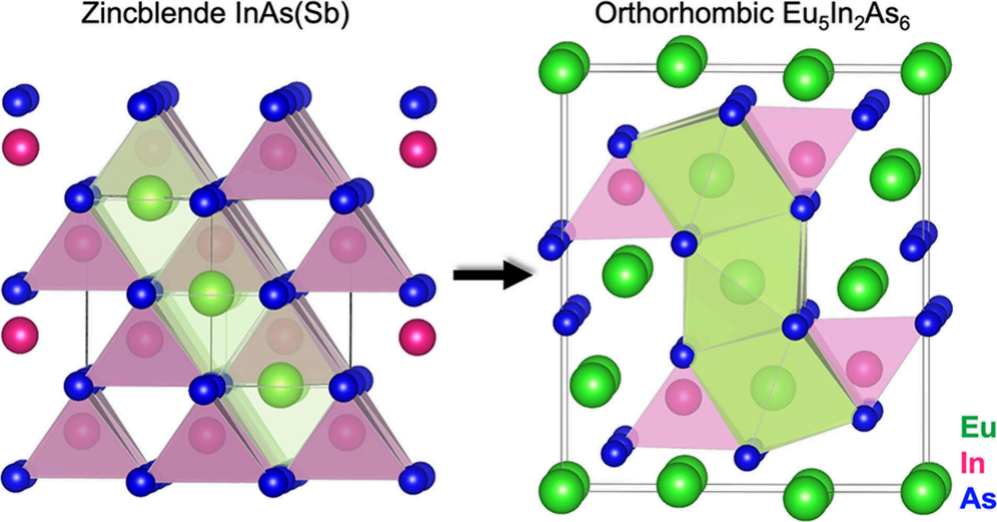

We demonstrate a topotactic transformation of zincblende InAs(Sb) nanowires into the Zintl phase Eu_5_In_2_As_6_ through a vapor–solid mutual exchange process involving Eu and In in molecular beam epitaxy. This conversion preserves the polyhedral coordination lattice of the parent InAs(Sb) structure while inducing orthorhombic symmetry in the product phase, Eu_5_In_2_As_6_, of which quasi-one-dimensional [InAs_3_]^6–^ chains with tetrahedral sites align along the ⟨110⟩ direction of zincblende structure. Local and global magnetic characterization identified two distinct antiferromagnetic phase transitions at approximately 7 and 16 K in Eu_5_In_2_As_6_ nanowires, potentially classified as altermagnetic phases. The versatility of the topotactic conversion of III–V semiconductor nanowires provides a platform for designing functional Zintl materials with tunable magnetic properties, making them promising candidates for spintronic applications.

III–V semiconductor nanowires (NWs) have emerged as fundamental building blocks in nanotechnology thanks to their exceptional electronic and optical properties. Indium arsenide (InAs) NWs, in particular, are notable for their high electron mobility and strong spin–orbit coupling, which make them ideal for advanced applications in electronics, photonics, and quantum computing.^1−4^ One significant application involves hybridizing InAs NWs with superconductors to realize Majorana zero modes under an external magnetic field. The strong spin–orbit coupling and Zeeman splitting in InAs NWs enable the formation of these exotic quasiparticles, which are promising for topological quantum computing thanks to their non-Abelian statistics and potential for fault-tolerant computation.^5−8^ Additionally, doping InAs NWs with small amounts of transition metals like manganese (Mn) and iron (Fe) induces ferromagnetic (FM) ordering, resulting in diluted magnetic semiconductors (DMS).^9,10^ These DMS materials are promising candidates for spintronic devices, where the electron’s spin degree of freedom is utilized for information processing and storage, potentially enhancing device functionality and efficiency.^11−13^

Recently, a topotactic growth mechanism using the rare-earth element europium (Eu) has been introduced to induce antiferromagnetic (AFM) ordering in InAs NWs. Specifically, solid-state mutual cation exchange has been used to convert wurtzite (WZ) InAs NWs into the Zintl phase Eu_3_In_2_As_4_, which exhibits AFM behavior.^14−16^ Thus, this innovative approach provides the means for the incorporation of magnetic properties into the as-grown III–V materials. In this study, we extend the method of topotaxy to zincblende (ZB) InAs(Sb) NWs, demonstrating their conversion into the Zintl phase Eu_5_In_2_As_6_, which orders antiferromagnetically below 17 K. The ability to transform the same semiconductor NWs into either FM or AFM materials through selective growth methods provides a versatile platform for designing spintronic devices with tailored magnetic properties. Such tunability could enable the development for future spintronic technologies.^17,18^

Vertical and reclining core NWs were grown by Au-assisted vapor–liquid–solid (VLS) molecular beam epitaxy (MBE) on (111)B and (001) InAs substrates, respectively. To obtain a ZB structure, we added a low flux of Sb after short growth of WZ InAs NWs in our previous work.^19^ It was demonstrated, however, that the WZ InAs transforms into Zintl-phase Eu_3_In_2_As_4_ shells via mutual-exchange topotaxy.^14^ Without WZ InAs stumps, ZB InAs_1–*x*_Sb_*x*_ NWs (Sb 5–7 atomic %) were grown for 1.5 h by opening both As and Sb shutters simultaneously under a high pressure As environment (Methods in SI). The ZB InAsSb cores are approximately 50 nm in diameter and 3–4 μm long. Subsequently, the In source was closed and cooled down, and Eu and As were evaporated for 2 h to form the Eu–In–As shell. Scanning electron microscopy (SEM) images of the resulting NWs on both substrates are shown in Figure 1a and 1b. Zoomed-in SEM images (Figure 1c and 1d) show a polycrystalline-like structure with pointed shapes along the core NW axis. It is worth noting that adding Eu to the MBE chamber did not impede the growth of III–V NWs; however, the purity and the resulting mobilities might be degraded.

**Figure 1:**
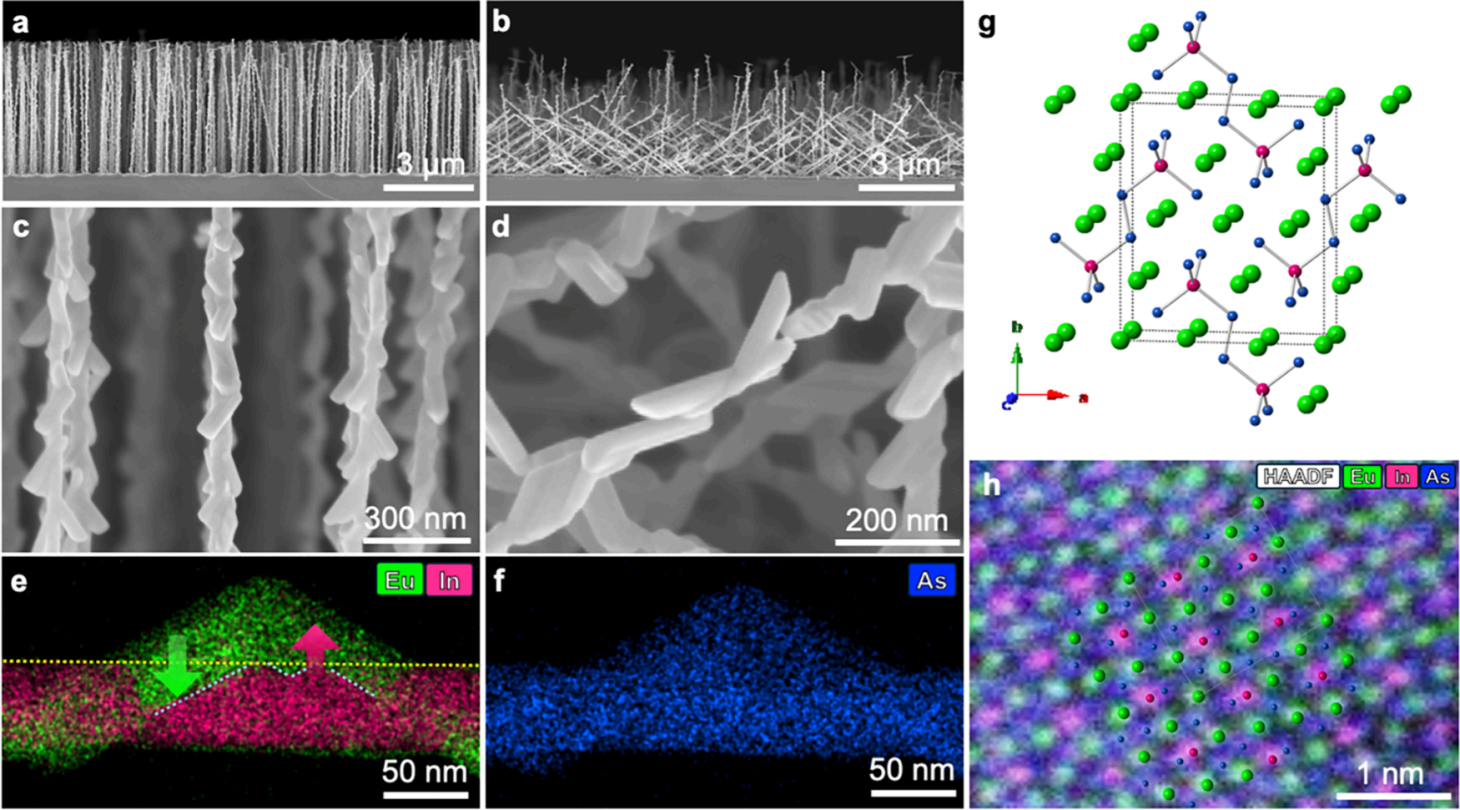
Exchange growth of the Zintl Eu_5_In_2_As_6_ shell on ZB InAsSb core NWs. (a, b) Cross-sectional SEM images of the Eu_5_In_2_As_6_ shell grown on ZB InAsSb core NWs, which were pregrown on (111)B and (001) InAs substrates, respectively. (c, d) Magnified SEM images of Eu_5_In_2_As_6_ NWs revealing grains with unique pointed shapes. (e) EDS maps of Eu (green) and In (magenta), showing their uniform distribution on either side of the core boundary. Green and magenta arrows indicate the mutual exchange of Eu and In atoms across the virtual boundary (yellow dashed line). (f) EDS map of As showing its uniform distribution across the core and shell. (g) Crystal structure of Zintl Eu_5_In_2_As_6_. (h) EDS map of Eu (green), In (magenta), and As (blue) atoms overlaid on the high-resolution high-angle annular dark-field (HAADF) STEM image, showing the crystal structure of Eu_5_In_2_As_6_ along the [001] zone axis.

We examined the NW’s stoichiometry and crystal structure by transmission electron microscopy (TEM). The crystallites grew both outward as a shell and inward penetrating into the NW core (Figures S01–S03) as we observed in Eu_3_In_2_As_4_ NWs.^14^ The crystalline structure of the shells in TEM images has a different morphology compared to those seen along the Eu_3_In_2_As_4_ NWs. Furthermore, energy-dispersive X-ray spectroscopy (EDS) analysis was performed to resolve the elemental composition. Mutual exchange of In from the core and Eu from the shell takes place across the boundary (yellow dashed line in Figure 1e) along the ZB NW, similar to the Eu_3_In_2_As_4_ crystallites on WZ NWs. The atomically sharp phase boundary (light-blue colored dashed line in Figure 1e) forms between the ZB core and the Eu–In–As shell (Figure S04). From the analysis of the relative EDS intensities (Figures S04–S05), we determined the stoichiometry of such shells to be Eu_5_In_2_As_6_ despite Sb intervention in both the core and shell. Eu_5_In_2_As_6_ grains can also form via mutual exchange in reclined NWs on (001) substrates (approximately 55° relative to the substrate normal). The large angle at which Eu and As atoms impinge on the surfaces of these reclined NWs enhances the mutual exchange process compared to vertical NWs.^20^ As a result, an axial junction of Eu_5_In_2_As_6_ grains and core is formed (Figure S06), making detailed investigation challenging. In the following discussion, we will focus solely on vertical NWs.

High-resolution scanning transmission electron microscopy (HRSTEM) demonstrated the perfectly ordered structure of the Eu_5_In_2_As_6_ crystallites (Figure S07). HRSTEM-EDS shown in Figure 1h allowed us to elucidate the material structure as another Zintl phase with the orthorhombic space group *Pbam*.^21^ Its unit cell is shown in Figure 1g as well as overlaid in Figure 1h. It includes two quasi-one-dimensional polyanionic chains of [InAs_3_]^6–^ aligned with the *c*-axis of the orthorhombic Eu_5_In_2_As_6_ phase. The double chains are allocated to [In_2_As_6_]^10–^ ribbons with As–As bonds, as described in ref^21 and 22^. The Eu atoms are arranged among the [In_2_As_6_]^10–^ ribbons in an intertwined pattern resembling the Greek letter lambda (λ), with each stroke containing five Eu^2+^ cations^22^ (Figure S07d). This coordination is also characteristic of the isostructural Zintl phases of Ca_5_Ga_2_As_6_ and Sr_5_In_2_As_6_.^21,23^ In a mechanistic picture, continuous chains of edge-sharing InAs tetrahedra in Eu_3_In_2_As_4_ are formed from the corner-sharing tetrahedra in WZ InAs, and split into isolated anionic chains during the Eu uptake. The corner-sharing tetrahedral coordination in ZB InAs, on the other hand, become the tetrahedral chains in Eu_5_In_2_As_6_ without a change of their sharing configuration throughout an influx of Eu.

In our previous work,^14^ the Zintl phase Eu_3_In_2_As_4_ was formed as a shell on underlying WZ InAs core via a mutual-exchange process. The formation of Eu_5_In_2_As_6_ differs from that of Eu_3_In_2_As_4_ in many respects. Eu_5_In_2_As_6_ was formed with a shape of grainy crystallites on ZB InAsSb NWs rather than forming a shell. The crystallites grow and elongate in specific directions, having angles of approximately 35°, 145°, and rarely 90° from the ⟨111⟩ direction (growth axis) of NWs, as indicated by blue arrows in Figures 2a–c. HRTEM was performed to investigate the interface between Eu_5_In_2_As_6_ and ZB InAsSb NWs. As shown in Figures 2d–i, three types of interfaces were found, designated as types A, B, and C, which denote different zone axes of Eu_5_In_2_As_6_. Fast Fourier transform (FFT) analysis for each type is shown in Figure S01. In type A (Figures 2d–e), the orientation between Eu_5_In_2_As_6_ and InAsSb is ⟨100⟩_*EuInAs*_ ∥ ⟨110⟩_*InAsSb*_, and the interfacial planes are (001)_*EuInAs*_ ∥ (001)_*InAsSb*_. For type B, the orientation relationship is ⟨010⟩_*EuInAs*_ ∥ ⟨112⟩_*InAsSb*_; however, the interfacial planes for type B are ambiguous. The Eu_5_In_2_As_6_ grains in both type A and type B have the same tiling angle and neighboring grains with another zone axis, ⟨110⟩_*EuInAs*_, which corresponds to a viewpoint near the midpoint between the *a*- and *b*-axes (Figures S02 and S03). For type C (Figures 2h–i), the orientation is ⟨001⟩_*EuInAs*_ ∥ ⟨110⟩_*InAsSb*_, and the interfacial planes are ⟨100⟩_*EuInAs*_ ∥ ⟨111⟩_*InAsSb*_. Eu_5_In_2_As_6_ crystallites of type C were rarely found. However, wing-shaped and ∼90°-tilted grains near the tip of the NW (the upper two arrows in Figure 2a) are equivalent to type C. Figures 2a and 2h have a rotating relationship of 90° around the NW growth direction, considering the morphological shape and crystallographic direction of the wing-shaped grain illustrated in Figure S08.

**Figure 2:**
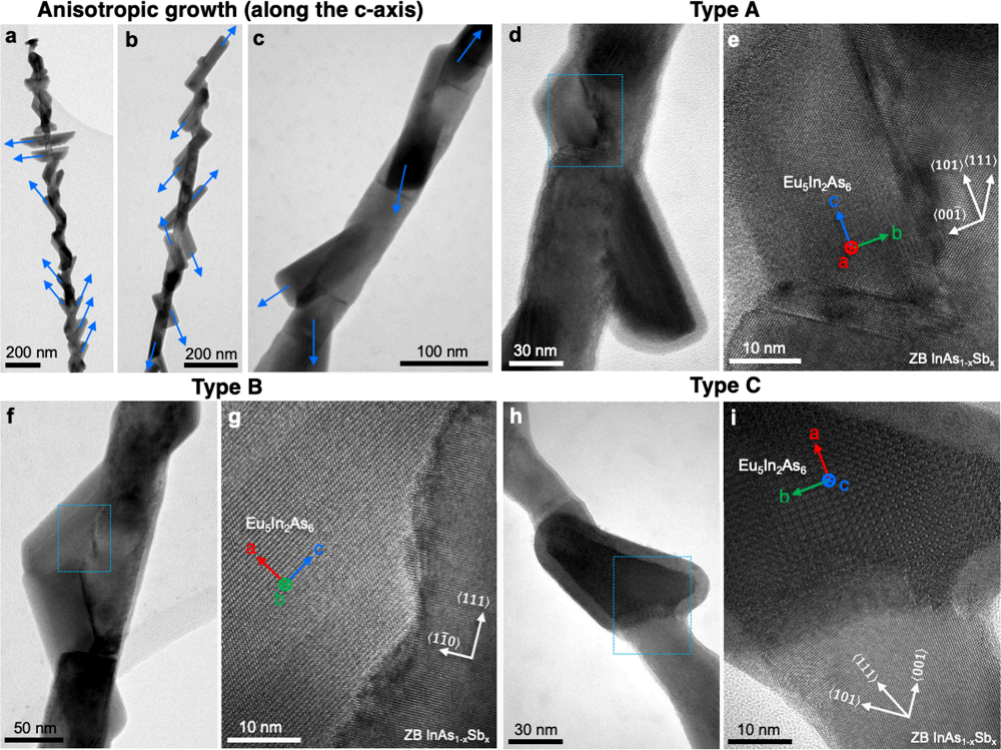
Anisotropic growth of the Zintl Eu_5_In_2_As_6_ shell along the *c*-axis. (a–c) TEM images of the as-grown Eu_5_In_2_As_6_ on InAsSb NWs. The blue arrows indicate crystallographic *c*-axis direction of Eu_5_In_2_As_6_, which forms angles of approximately 35°, 145°, and rarely 90°, relative to the ⟨111⟩ direction of the core NWs. (d) Enlarged TEM image of Type A crystal. (e) HRTEM image of the area marked by a blue rectangle in (d), showing the interface in the ⟨100⟩*_EuInAs_* and ⟨110⟩_*InAsSb*_ zone axes. (f) Enlarged TEM image of Type B crystal. (g) HRTEM image of the area marked by a blue rectangle in (f), showing the interface in the ⟨010⟩*_EuInAs_* and ⟨112⟩*_InAsSb_* zone axes. (h) Enlarged TEM image of Type C crystal. (i) HRTEM image of the area marked by a blue rectangle in (h), showing the interface in the ⟨001⟩*_EuInAs_* and ⟨110⟩_*InAsSb*_ zone axes. This corresponds to the wing-shaped crystallites in (a) (the first and second blue arrows from the top). Respective fast Fourier transform (FFT) images used to identify the crystal structures and zone axes are shown in Figure S01.

All types of Eu_5_In_2_As_6_ crystallites exhibit anisotropic growth along the *c*-axis and have selective tilting angles with respect to the InAsSb NWs since the InAs tetrahedra in the NWs are the structural motifs that reappear in the [InAs_3_]^6–^ tetrahedral chains in Eu_5_In_2_As_6_. In Figure S09 (zone axis [110] of InAs, type A and C), the [InAs_3_]^6–^ chain consists of continuous corner-shared InAs tetrahedra aligned along the *c*-axis. We observed that the *c*-axis of Eu_5_In_2_As_6_ aligns parallel to any of the equivalent ⟨110⟩ directions of InAsSb, as shown in Figures 2e and 2i. Hence, the interface between ZB and Eu_5_In_2_As_6_ in type B cannot be observed in the zone axis [112] of the InAs tetrahedra (seemingly WZ) since both crystal structures overlap along the viewing direction. For the same reason, Eu_5_In_2_As_6_ crystallites with ⟨110⟩_*EuInAs*_ or at some midpoints between the a- and b-axes were found. This tendency is maintained in an exceptional Eu_5_In_2_As_6_ formation. In Figure 3, a few NWs grew along the ⟨110⟩ direction and displayed smoothly coated Eu_5_In_2_As_6_ shells, as observed in both SEM and TEM analyses. These extraordinary NWs have potential for application of well-defined InAs-Eu_5_In_2_As_6_ core–shell NWs and possibly the axial growth of Eu_5_In_2_As_6_ NWs.

**Figure 3:**
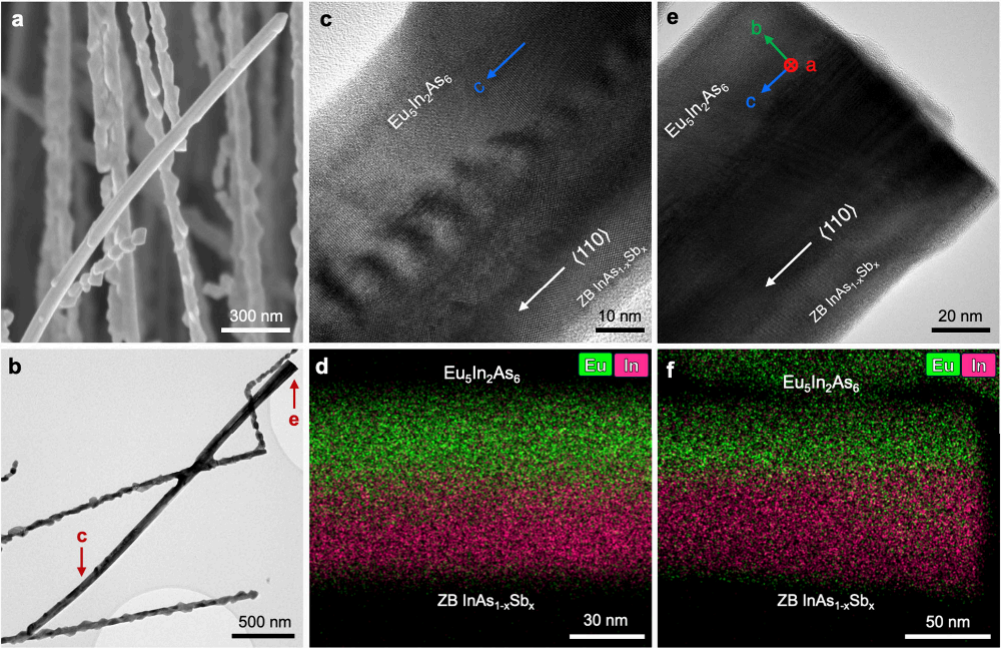
Smoothly coated Eu_5_In_2_As_6_ shell on ⟨110⟩-oriented ZB InAsSb core NWs. (a) SEM and (b) TEM images of as-grown Eu_5_In_2_As_6_ on ZB InAsSb NWs grown exceptionally along the ⟨110⟩ direction. Enlarged TEM images near the tip (c) and the bottom (e), indicated by the red arrows in (b), show that the *c*-axis of Eu_5_In_2_As_6_ is parallel to the ⟨110⟩ direction of the ZB core. (d, f) EDS elemental maps of Eu (green) and In (magenta) from (c) and (e), respectively. Only these two elements are shown to emphasize a different core–shell configuration. Detailed EDS and FFT analyses are provided in Figures S10–S13.

Topotaxial mutual-exchange growth involves the structural similarity between the parent and resulting crystals, where cations such as Eu and In mutually exchange in the As skeleton.^14^ Therefore, it is necessary to examine the crystallographic resemblance between Zintl Eu_5_In_2_As_6_ and ZB InAs(Sb), as well as to understand the processes of transformation and rearrangement. As our previous studies^19^ have shown, Eu atoms are highly reactive^24^ and can readily permeate into the ZB InAs(Sb) matrix. After Eu intrusion, the corner-sharing InAs tetrahedra in the ZB structure are maintained, as mentioned above; however, the tetrahedra are both corner- and edge-shared with Eu^2+^ octahedra. Half of them are alternately inverted, and Eu^2+^ octahedra are intercalated three-dimensionally within the ZB matrix. This distribution of Eu atoms resembles that of Eu in the (EuIn) As mosaic structure (Figure S14). As a result, the InAs tetrahedra in the ZB structure are transformed into the [In_2_As_6_]^10–^ double chains along the *c*-axis in the orthorhombic Eu_5_In_2_As_6_ structure. Common with the transition of WZ InAs into Eu_3_In_2_As_4_ previously reported by us^14^ is the topotaxial character of the transformation. The As framework of the parent phase is maintained in the sense that each As atom is a node in the polyhedral network of the product phase. Here, the cubic symmetry elements of the InAs(Sb) parent phase afford the resulting phase to have a specific symmetry resulting in a unique phase and composition. For each of the nodes in the As framework, there is a *one-to-one* relationship between the site in the parent phase and the site in the product phase (Figure 4). The polyhedral symmetry spanned by the node sites is maintained in a slightly distorted form. The distance between As sites in the Eu_5_In_2_As_6_ lattice is dilated on average by as little as 2% when compared with the As sublattice in the cubic InAs(Sb). Figure 4a shows the cubic InAs in a ⟨110⟩ viewing direction with its two types of tetrahedral sites T+ and T–, of which one is occupied by In in the parent phase. Chains of T+ and T– sites in InAs along the ⟨110⟩ correlate with the [InAs_3_]^6–^ chains in Eu_5_In_2_As_6_ along [001] seen in Figure 4b. Initially unoccupied octahedral sites on InAs {111}-planes are filled by Eu to form the planes of alternating EuAs_6_ octahedra in the Eu_5_In_2_As_6_ lattice. A second type of Eu site is coordinated in prismatic geometry, originating from tetrahedral sites in the InAs. The alignment of the [InAs_3_]^6–^ chains with tetrahedral sites along the InAs ⟨110⟩ directions provides the intuitive understanding for the observed orientational relationships where the *c*-axis of Eu_5_In_2_As_6_ coincides with any of the six equivalent ⟨110⟩ directions of the parent phase. It further supports such an alignment that the top-view pattern of Eu_5_In_2_As_6_ crystallites (Figure S15) manifests a 3-fold symmetry.

**Figure 4:**
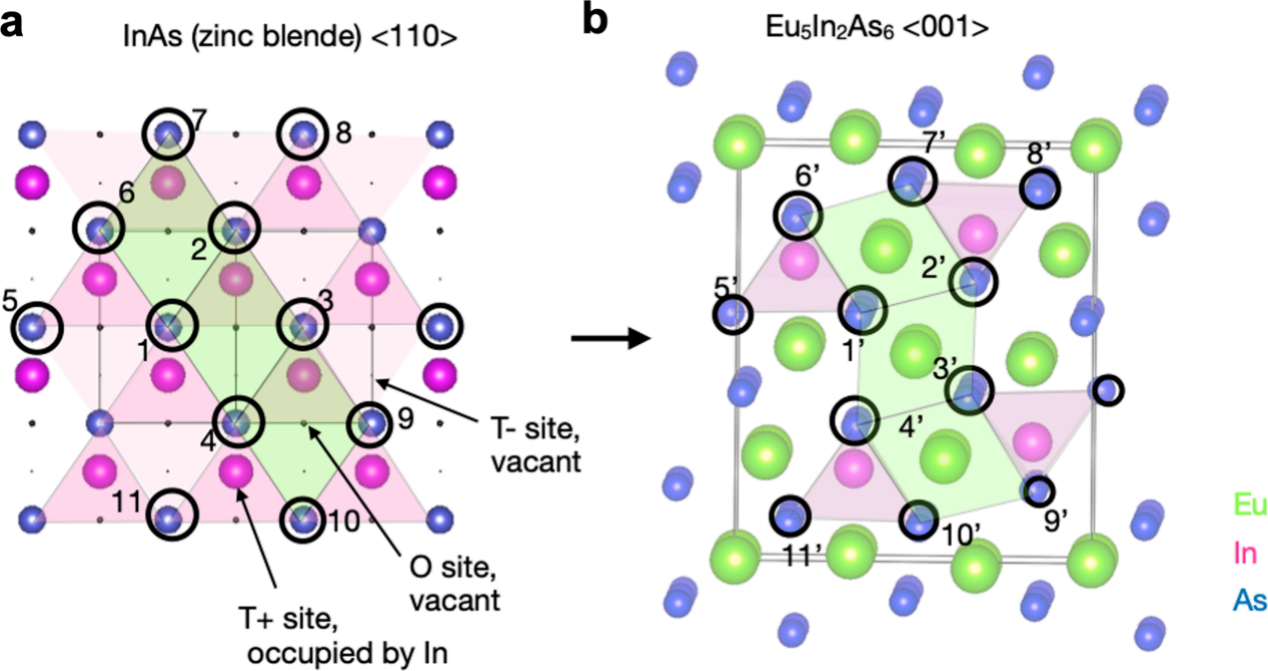
Topotaxial transformation of zincblende InAs into orthorhombic Eu_5_In_2_As_6_. (a) Crystal structure of cubic InAs in ⟨110⟩ viewing direction. Tetrahedral sites T+ and T– are filled in pink, and octahedral sites are in green. (b) Crystal structure of Eu_5_In_2_As_6_ in [001] viewing direction. Site nodes of the As framework map onto one another with a linear expansion of the lattice by about 2% in total. Layers of octahedrally coordinated Eu correspond to layers formed by filling vacant octahedral sites in the InAs on {111} planes (e.g., path 1–2–3–4 vs 1′–2′–3′–4′). Corner-sharing InAs tetrahedra aligned and the related InAs tetrahedra forming [InAs_3_]^6–^ chains along [001] emerge from the tetrahedral T+ and T– sites along a ⟨110⟩ direction in the cubic InAs (paths 1–5–6 vs 1′–5′–6’ and 2–7–8 vs 2′–7′–8′).

Mechanistically, the As(Sb) sublattice in InAs(Sb) forms a nonrigid framework for the transformation. The topotactic conversion occurs under indium-deficient conditions (i.e., without any external indium supply). We hypothesize that the transformation is initiated by a codiffusion of Eu and In through vacant sites in the framework while maintaining charge neutrality. The gradient in chemical potential for the diffusion process is maintained at the interface to the gas-phase by the supply of Eu but also As for the consumption of In. Unlike in the mosaic structure reported before,^19^ where indium was continuously supplied, here the indium shortage promotes In diffusion out of the InAs NW core, resulting in the formation of Eu_5_In_2_As_6_ grains. In contrast, the external indium supply prevents the formation of Eu_5_In_2_As_6_ and embeds EuAs sheets within polycrystalline InAs. Therefore, the critical indium concentration is a key factor in the topotaxial formation of Eu_5_In_2_As_6_.

We used a combination of local scanning superconducting quantum interference device (SQUID) measurements and global SQUID magnetometry in a commercial magnetic property measurement system (MPMS). In local scanning SQUID of individual NWs we did not detect DC magnetization up to 1 mΦ_0_ at the location of the Eu_5_In_2_As_6_ NWs alongside finite AC susceptibility, χ, shown in Figure 5a and b, respectively. The absence of a DC signal put a strict limit on the strength of magnetization associated with a ferromagnetic order down to our sensitivity, but the strength of the measured paramagnetic signal signified the presence of magnetic moments. We further examined the temperature dependence of the AC susceptibility signal across the NWs. The paramagnetic signal from two different NWs, shown in Figure 5c (Figure S16 in SI), exhibited a nonmonotonic behavior. We found two local maxima, one at 7 K and a slightly fainter one at 15–17 K. The cusp profile and lack of DC magnetization indicated the two transitions in the magnetic order are into antiferromagnetic phases. Interestingly, even though the intensity of the measured signal from each NW was slightly different, the magnetic phase transitions were consistent.

**Figure 5:**
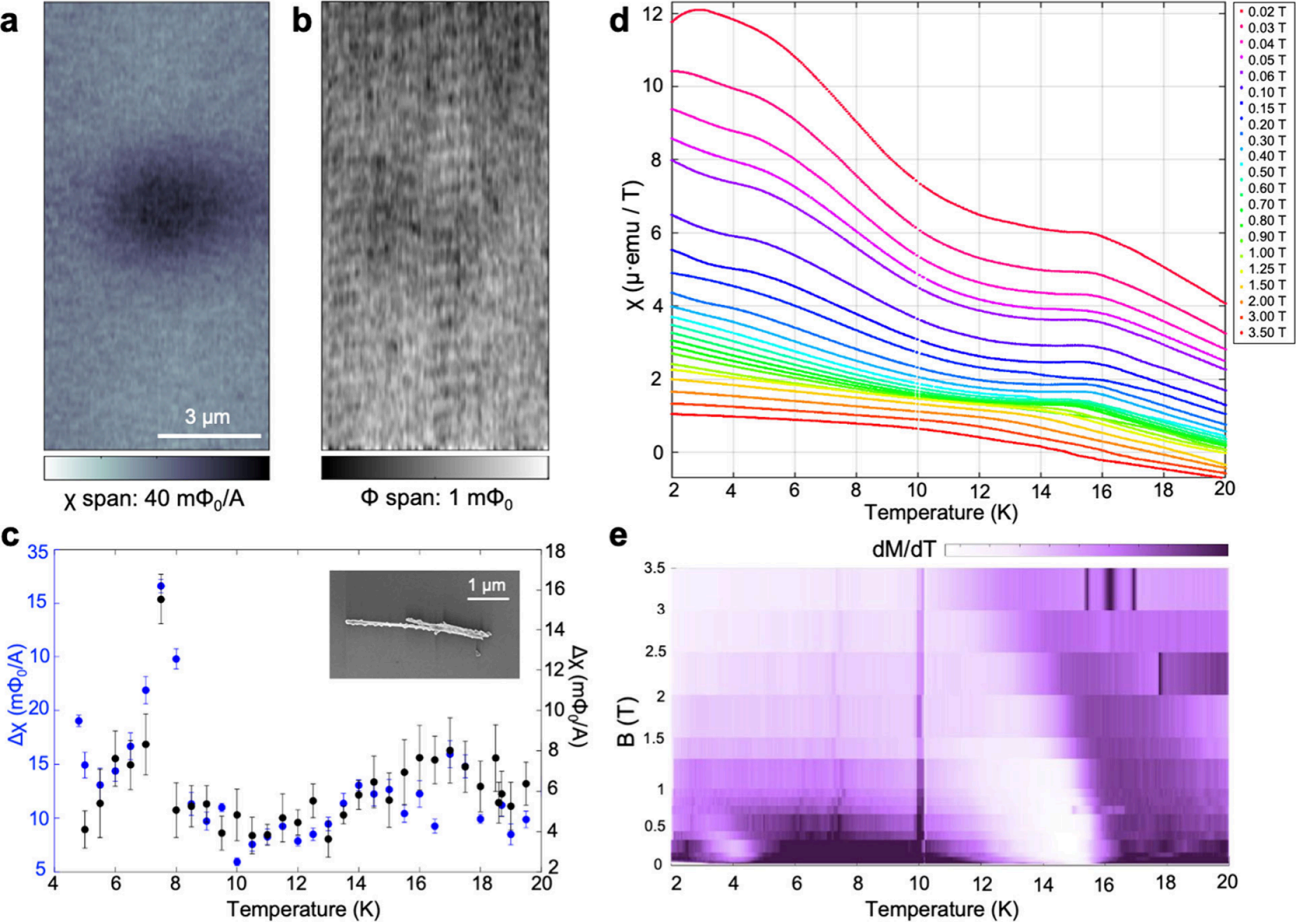
Magnetic characterization of the Zintl Eu_5_In_2_As_6_ shell along the *c*-axis. (a) Local AC susceptibility and (b) DC magnetism maps of Eu_5_In_2_As_6_ NWs. (c) AC susceptibility of the NWs versus temperature, showing changes in trend at about 7 K and 15–17 K. The error bars are the standard deviation of pixel values in each relevant area in the vicinity of the two maximum local signals (Figure S16). (d) Global susceptibility (c = B/H) measurements of the dispersed Eu_5_In_2_As_6_ NWs with a SQUID in a MPMS showing the evolution of two Néel transitions at about 6 and 16 K. (e) Plot of dM/dT, where two Néel transitions appear, presented in false color from (d).

Therefore, to improve the signal-to-noise and better resolve the magnetic phase diagram of the Eu_5_In_2_As_6_ NWs in temperature as well as in a magnetic field, we measured the global magnetization of millions of NWs harvested and deposited over a nonmagnetic Si/SiO_2_ substrate (Methods in SI) for MPMS. Individual magnetization curves taken along temperature sweeps at various applied magnetic fields are shown in Figure 5d. At low magnetic fields, we resolved the two cusps in the magnetization at about 6 and 16 K. This result is consistent with that obtained from bulk Eu_5_In_2_As_6_.^22^ Both shifted gradually to lower temperatures as the applied field increased. The resulting magnetic field-temperature phase diagram is shown in Figure 5e by plotting in false color the derivative of the magnetization with temperature, dM/dT, to enhance the transition temperature. It thus hosts two distinct antiferromagnetic phases whose exact spin structures call for further investigation. Intriguingly, given the Eu substructure within Zintl Eu_5_In_2_As_6_ those magnetic orders are more accurately classified as altermagnetic.^25−27^

In this work, we realized Zintl phase Eu_5_In_2_As_6_ NWs, which present clear AFM ordering, through a vapor–solid topotactic mutual exchange in MBE. This approach builds on our recently introduced method for growing Zintl Eu_3_In_2_As_4_ NWs.^14^ Our findings suggest that the ZB or WZ polytypes of indium pnictide core NWs could yield separate orthorhombic Zintl compounds, X_5_In_2_Y_6_ or X_3_In_2_Y_4_ (X = Sr, Ba, Yb; Y = As, Sb), each crystallizing in the *Pbam* or *Pnnm* space group, respectively. Furthermore, since ZB is the prevalent bulk structure in III–V crystals, our observation of topotactic conversion of ZB NWs may open the path to the application of this methodology to convert III–V substructure into Zintl films, potentially unlocking new possibilities in materials design and device integration.

## Supporting Information Available

Detailed experimental methods and additional structural, compositional, and magnetic characterization data of Eu_5_In_2_As_6_ NWs. These data feature SEM, HR-TEM, STEM-HAADF, EDS elemental mapping, schematic illustrations, and results from scanning SQUID measurements (PDF)
