# Supplementary material for: Topotactic Growth of Zintl Phase Eu5In2As6 Nanowires with Antiferromagnetic Behavior
Source: Nano Lett. 2025 Mar 11;25(18):7292–7. doi: 10.1021/acs.nanolett.5c00008 (PMC12063170; doi:10.1021/acs.nanolett.5c00008)
Supplement: Supplementary file 1 — nl5c00008_si_001.pdf [file nl5c00008_si_001.pdf]

SUPPLEMENTARY INFORMATION

Topotactic growth of Zintl phase $Eu_5In_2As_6$ nanowires with antiferromagnetic behavior

Man Suk Song,[1] Lothar Houben,[2] Nadav Rothem,[3] Ambikesh Gupta,[1] Shai Rabkin,[3] Beena Kalisky,[3] Haim Beidenkopf,[1]* and Hadas Shtrikman[1]*

[1]Department of Condensed Matter Physics, Weizmann Institute of Science, Rehovot 7610001, Israel

[2]Department of Chemical Research Support, Weizmann Institute of Science, Rehovot, 7610001, Israel

[3]Department of Physics and Institute of Nanotechnology and Advanced Materials, Bar-Ilan University, Ramat Gan, 5290002, Israel

Methods

$Eu_5In_2As_6$ NW growth

Zintl $Eu_5In_2As_6$ grains were topotaxially grown on the surface of ZB InAs NWs. As a preliminary step, vertical and reclining $InAs_{1-x}Sb_x$ NWs were grown by MBE (RIBER-32 CBE) on (111)B and (001) InAs substrates, respectively, using the Au-assisted VLS techniques, as described in previous work[1–4]. $InAs_{1-x}Sb_x$ NWs (Sb 5–7 atomic %) were grown for 1.5 hours by opening both As and Sb shutters simultaneously under a high pressure As environment. In and Sb temperatures (and fluxes) were 720°C (1.1×10^{-7} torr) and 425 °C (1.7×10^{-7} torr), respectively. The growth temperature was maintained at the typical for the growth of InAs NWs temperature (410 °C). To initiate the topotaxial cation exchange, the Eu shutter was opened as the In cell was cold and its shutter closed at the As flux was maintained. A 15-min pause for adjusting the cell temperatures followed the $InAs_{1-x}Sb_x$ NW growth. Right after opening of the Eu shutter, the substrate temperature was

ramped to a temperature 470 °C at a rate of 10 °C min^{-1}. The topotaxial exchange growth was maintained for 2 h. The temperatures (and fluxes) of Eu and As were 450 °C (3.8×10^{-8} torr) and 222 °C (1.8×10^{-6} torr), respectively. The substrate manipulator in the MBE system was maintained at the RIBER's so-called "standard epitaxy position" during both steps, without any adjustments.

Microscopy

The Zintl $Eu_5In_2As_6$ NWs were characterized by field-emission SEM (Zeiss Supra-55, 3 kV, working distance of ~4 mm) and TEM (Thermo Fisher Scientific Talos F200X, 200 kV). The EDS composition data and mapping images were obtained by TEM with an attached detector that is identical to the one used in scanning transmission electron microscopy (STEM).

HRSTEM images and analytical EDS maps were acquired in a double-aberration-corrected Themis-Z microscope (Thermo Fisher Scientific Electron Microscopy Solutions) at an accelerating voltage of 200 kV. The STEM images were recorded with a Fischione Model 3000 detector and a Thermo Fisher Scientific bright field detector. The EDS hyperspectral data were obtained with a Super-X SDD detector and quantified with Velox software (Thermo Fisher Microscopy Solutions), version 3.13, through background subtraction and spectrum deconvolution. The STEM images were obtained with an electron probe with a convergence angle of 21 mrad and a primary beam current of less than 50 pA; the EDS maps were recorded at a beam current of 200 pA.

SQUID

We used scanning SQUID microscopy to search for magnetic signals from the NWs. A SQUID converts magnetic flux to voltage, allowing a sensitive detection of magnetic fields[5]. The planar SQUID, in the scanning configuration, allows the mapping of the static magnetic landscape and the local susceptibility[6]. The sensitive area of the SQUID used in this work—the pickup loop—has a diameter of 1.5 μm. Local susceptibility was measured using an on-chip coil to apply a magnetic field (the field coil, operated in this work at ~kilohertz and ~Gauss), whereas the pickup loop records the local response to the applied magnetic field. The positive signal in our data indicates a paramagnetic response. These measurements of susceptibility were plotted in units of Φ_0 normalized by the sensitive area and current in the field coil.

We then performed these measurements as a function of temperature between 5 and 20 K to characterize the magnetic behaviour of the NWs. We later plotted the data as the inverse of the change in susceptibility.

In addition, the commercial SQUID magnetometer (MPMS3, Quantum Design) was also used to measure the massive $Eu_5In_2As_6$ NWs. To eliminate any possible magnetic contribution from the InAs substrate, we harvest the NWs by sonicating them for a few seconds in a solvent. We then disperse them over a Si/SiO_2 substrate (5×6 mm^2) by depositing the suspension and drying it drop by drop to exhaust all the NWs available[7]. This sample was inserted into a straw and mounted in MPMS3. The global magnetic measurement was carried out in the temperature range between 2 and 20 K by varying the magnetic field from 0.02 to 3.50 T.

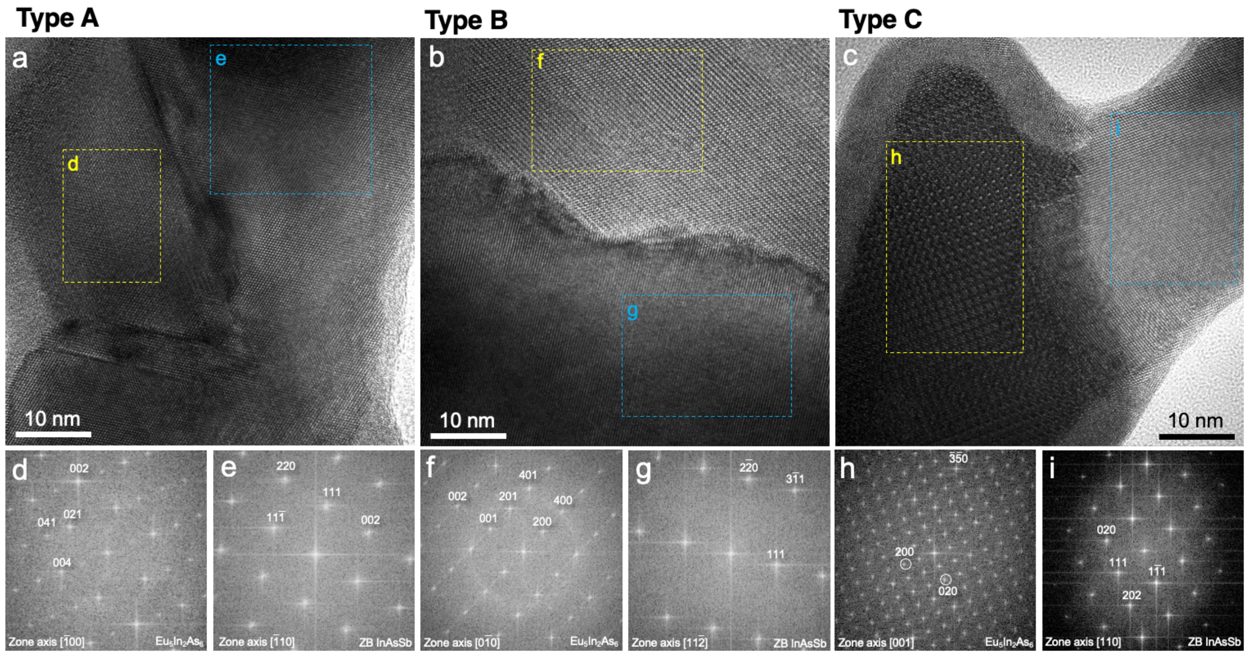

Figure S01 HR-TEM and FFT analyses of Zintl $Eu_5In_2As_6$ grains for Type A, B, and C. (a–c) Magnified HR-TEM images in Figures. 2e, 2g, and 2i, respectively. (d, f, g) FFT patterns of the yellow dashed rectangular areas in (a–c), demonstrating $Eu_5In_2As_6$ along the [-100], [0-10], and [001] zone axes, respectively. (e, g, h) FFT patterns of the blue dashed rectangular areas in (a–c), demonstrating ZB InAs(Sb) in the [-110], [11-2], and [110] zone axes, respectively.

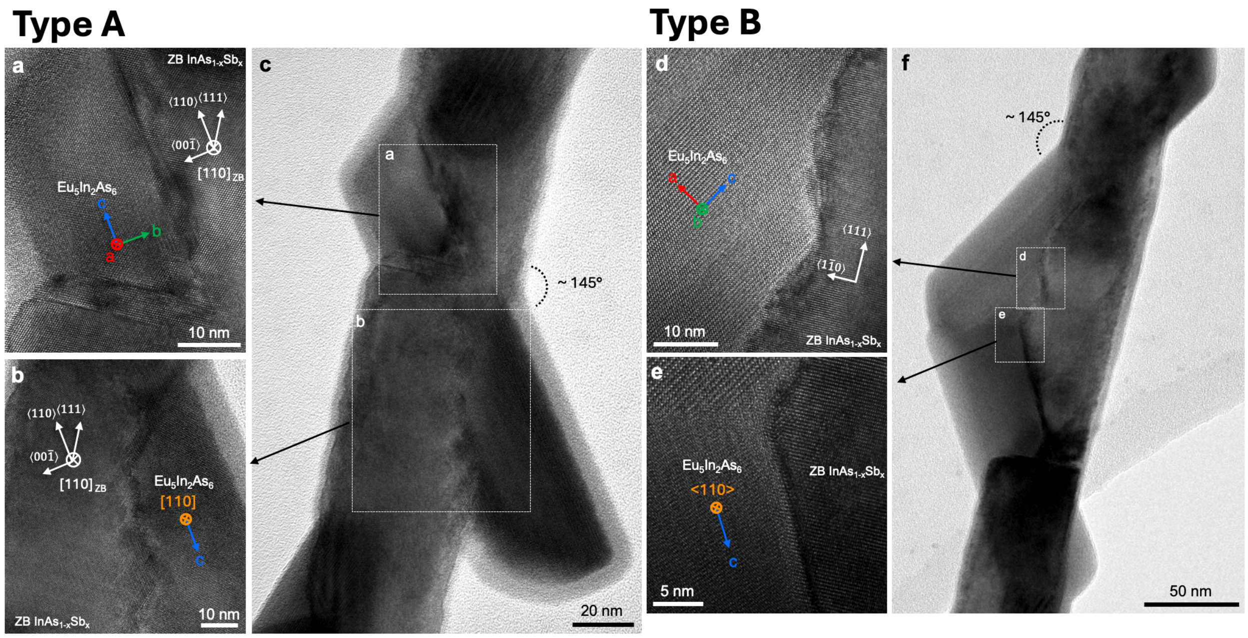

Figure S02 $Eu_5In_2As_6$ grains along the [110]. (a–b) Magnified HR-TEM images of the interface of Type A between $Eu_5In_2As_6$ and InAsSb, taken from the dashed white rectangular areas in (c). (c) Low-magnification TEM image of $Eu_5In_2As_6$ grains on a ZB NW, corresponding to Figure 2d in the main text.

(d–e) Magnified HR-TEM images of the interface of Type B between $Eu_5In_2As_6$ and InAsSb, taken from the dashed white rectangular areas in (f). (f) Low-magnification TEM image of $Eu_5In_2As_6$ grains on a ZB NW, corresponding to Figure 2f in the main text.

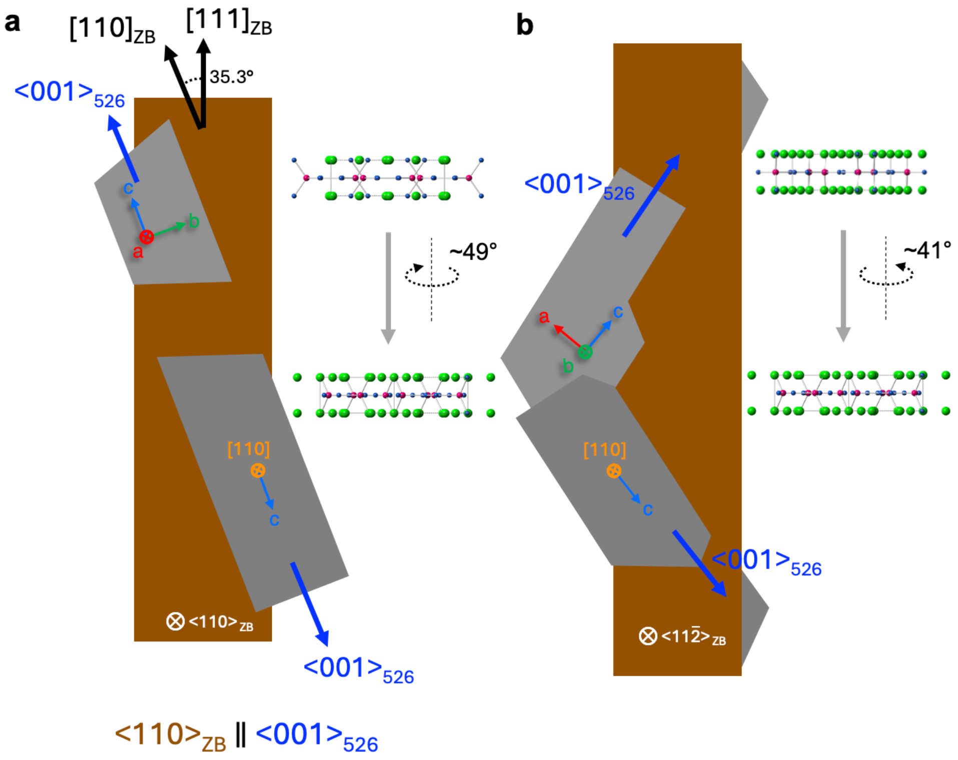

Figure S03 Schematics illustrating the crystallographic directions for each material along the identical zone axes of the ZB structure: (a) $<110>_{ZB}$ and (b) $<11\text{-}2>_{ZB}$, based on Figures 2d and 2f in the main text, respectively. These schematics highlight the orientation relationships between the $Eu_5In_2As_6$ grains and the InAsSb NW cores, and the coexistence of another $Eu_5In_2As_6$ grain along the [110] in different zones.

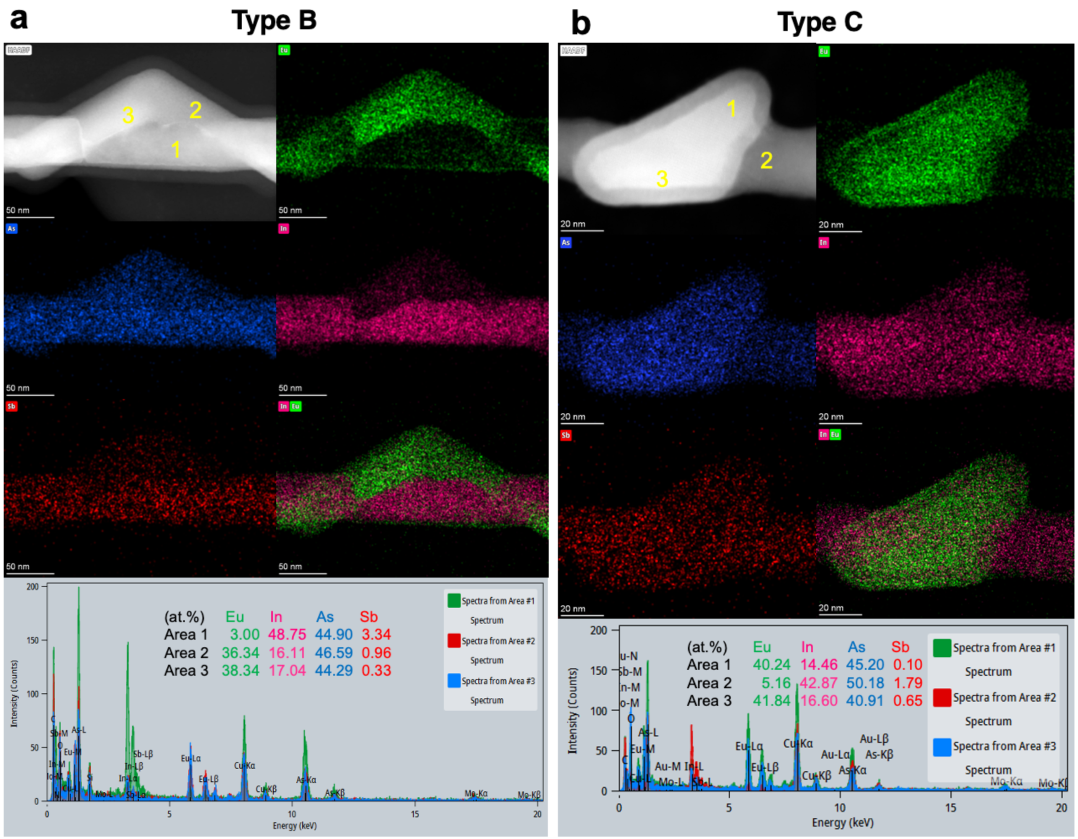

Figure S04 Elemental composition of a $Eu_5In_2As_6$ grain and ZB InAsSb core in (a) Type B and (b) Type C, corresponding to Figures 2f and 2h in the main text, respectively. Top: The $Eu_5In_2As_6$ and InAsSb areas were selected in HAADF STEM images for EDS measurement. EDS elemental maps show the distribution of Eu, In, As, Sb, and a composite map combining Eu and In. **Bottom:** Cumulative EDS spectra and their quantification extracted from the designated areas–Area 1, Area 2, and Area 3–in both types.

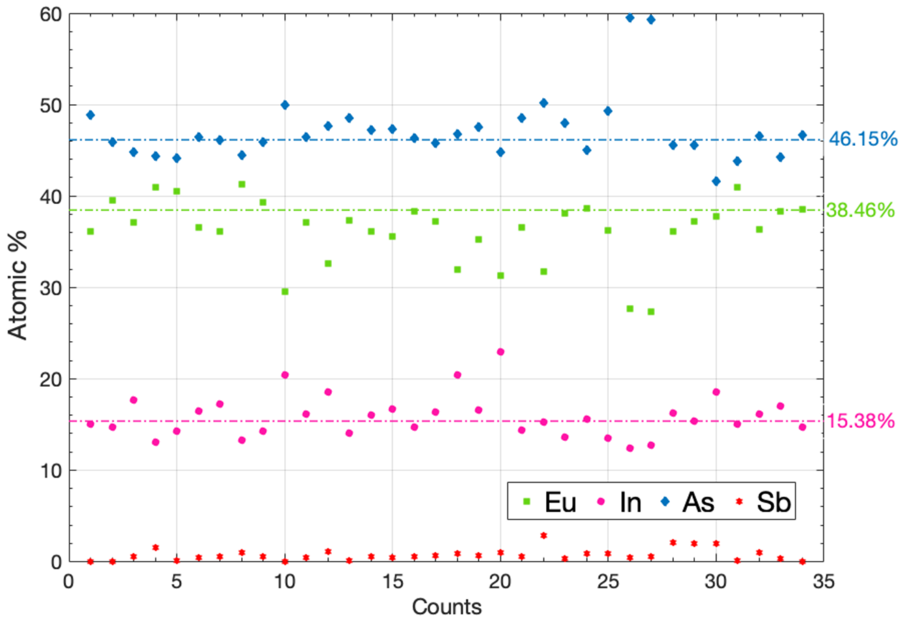

Figure S05 EDS atomic percentage distribution of Eu (green square), In (pink circle), As (blue diamond), and Sb (red star) for selected $Eu_5In_2As_6$ grains was measured by STEM-EDS. The three dash-dotted guidelines represent the ideal stoichiometric composition of $Eu_5In_2As_6$.

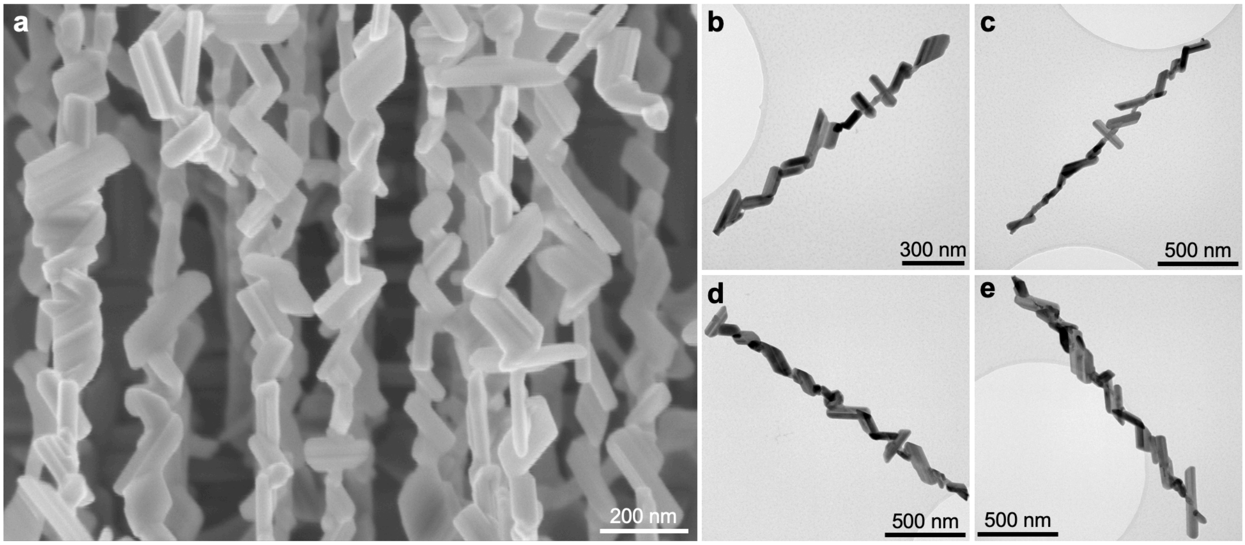

Figure S06 Intense mutual exchange in reclined NWs on (001) substrates. (a) Top-view SEM image and (b–e) TEM images of reclined $Eu_5In_2As_6$ NWs.

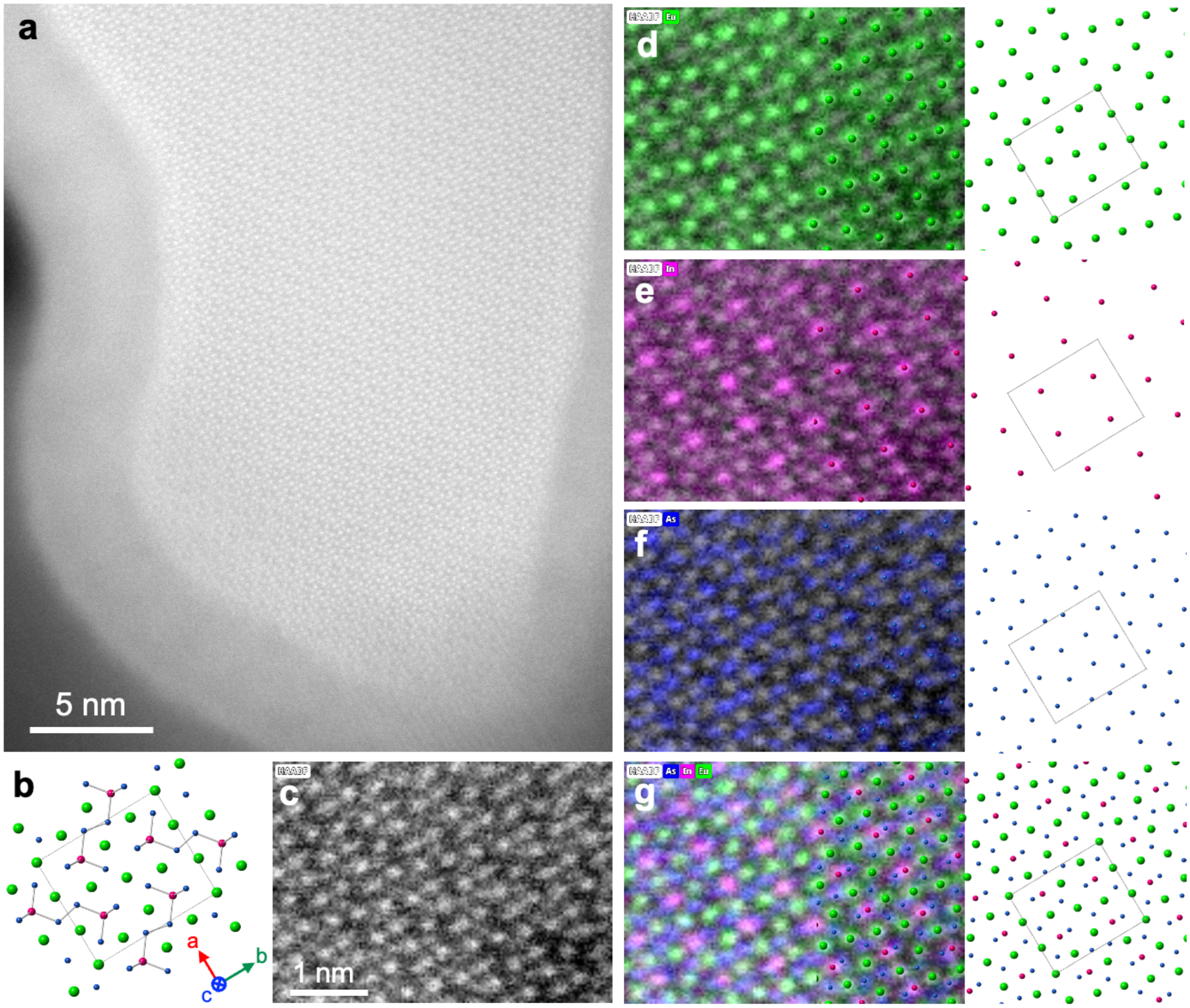

Figure S07 (a) STEM-HAADF image of a type C $Eu_5In_2As_6$ grain. (b) Crystal structure of Zintl $Eu_5In_2As_6$. (c) Atomic-resolution STEM-HAADF image clearly shows the crystalline structure of $Eu_5In_2As_6$ along the <001> zone axis. (d–g) Corresponding EDS elemental maps of Eu, In, As, and the combined map of all three elements from (c), respectively. An atomic model is continuously overlaid on the right side. The dash-lined rectangle indicates a 1×1 unit cell of $Eu_5In_2As_6$ along the <001> zone axis, as depicted in (b).

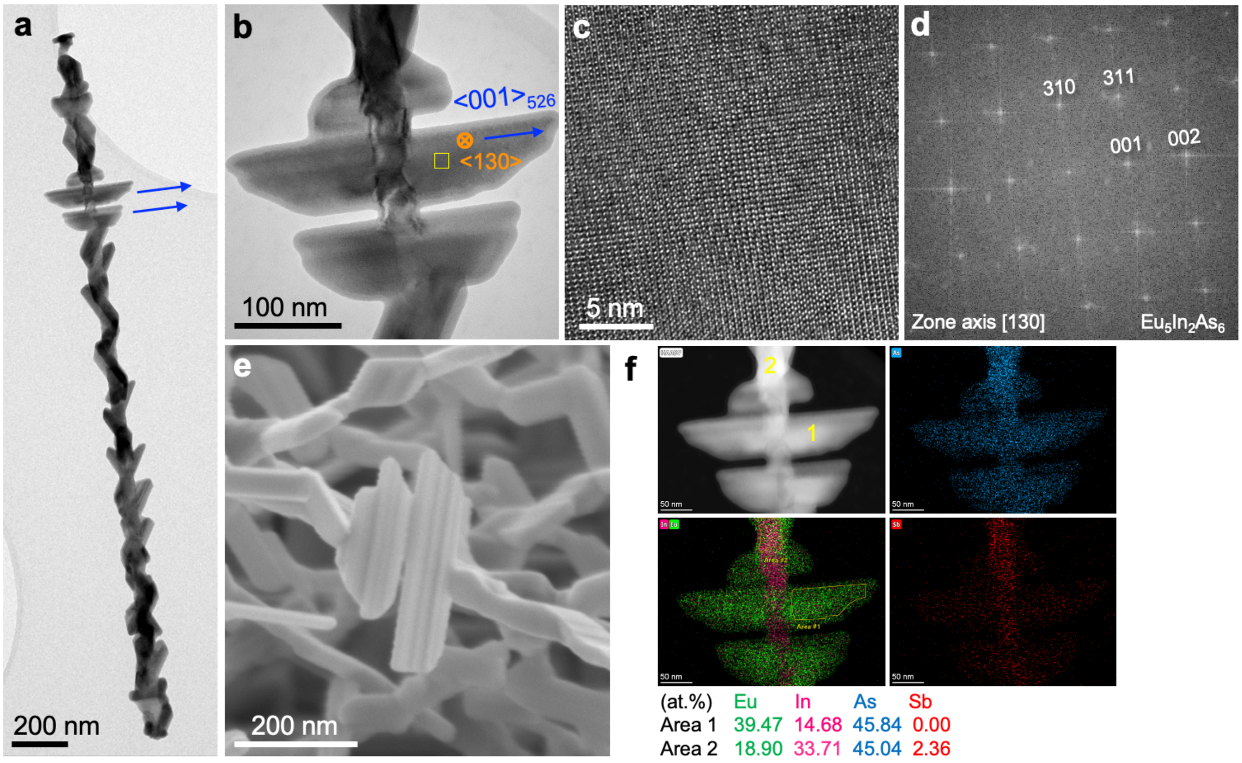

Figure S08 Wing-shaped type C $Eu_5In_2As_6$ grains. (a) TEM image of an $Eu_5In_2As_6$ nanowire. (b) Enlarged TEM image of the wing-shaped type C $Eu_5In_2As_6$ grains extending along the c-axis. (c) Further enlarged HRTEM image of the area indicated by the yellow square in (b). (d) FFT pattern from (c), showing the [130] zone axis of $Eu_5In_2As_6$. (e) A 45°-tilted SEM image of the wing-shaped grains. (f) HAADF (top left) and EDS elemental maps of As (top right), Sb (bottom right), Eu and In (bottom left) from (b). EDS spectra quantification for Areas 1 and 2 is shown below.

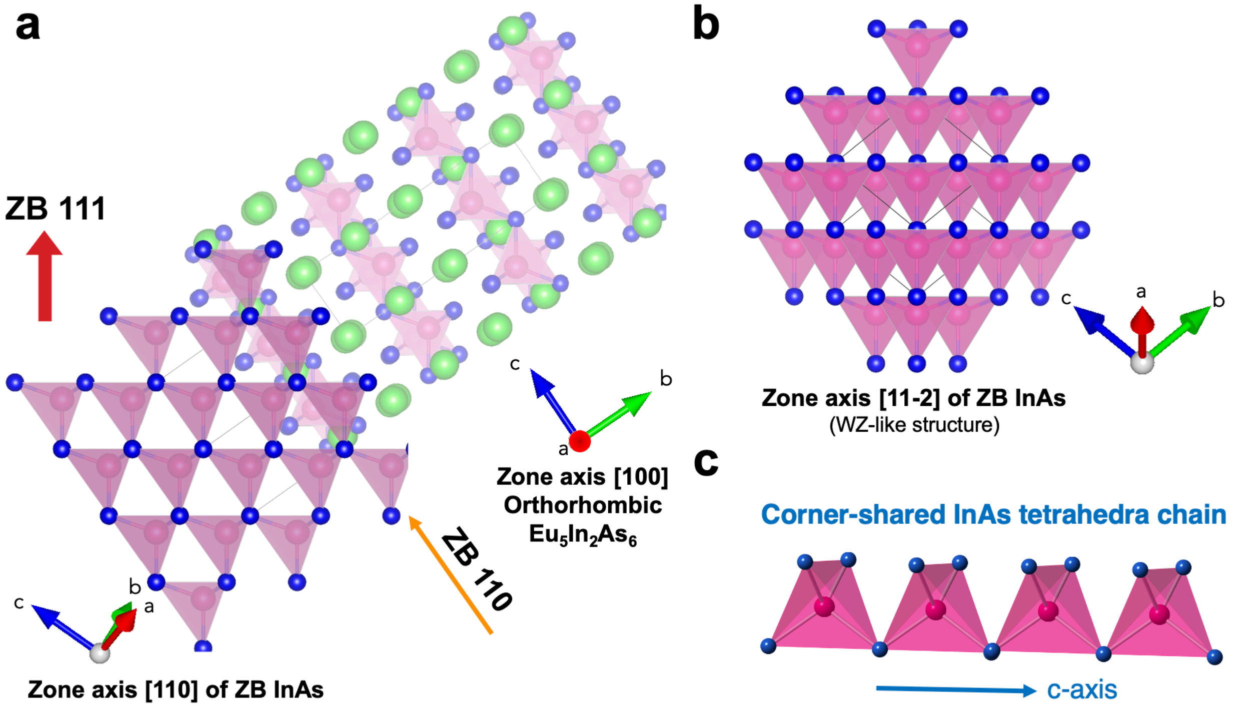

Figure S09 (a) Atomistic model for the type A (and C) interface along the respective viewing directions. The left model represents a ZB InAs NW, growing along the <111> direction (upward). The orange arrow indicates the <110> direction of the boundary line, parallel to the c-axis of orthorhombic $Eu_5In_2As_6$. In $Eu_5In_2As_6$, the $[InAs_3]^{6-}$ chain consists of continuous corner-shared InAs tetrahedra aligned along the c-axis. (b) Atomistic model of InAs viewed along the [11-2] direction (type B), which is at a 30° relative to the <110> direction. From this perspective, the structure seemingly looks WZ InAs. (c) Schematic illustration of the corner-shared $[InAs_3]^{6-}$ chain in the Zintl phase $Eu_5In_2As_6$.

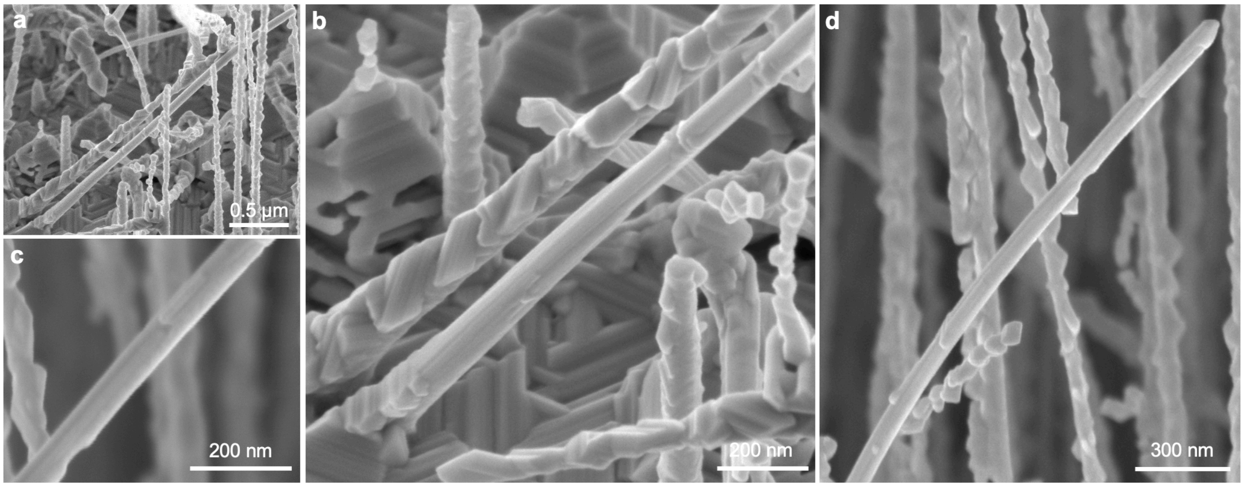

Figure S10 (a–d) SEM images of $Eu_5In_2As_6$ smoothly coated NWs. Notably, the two NWs seen in (a) and (b) have clearly different surface morphologies.

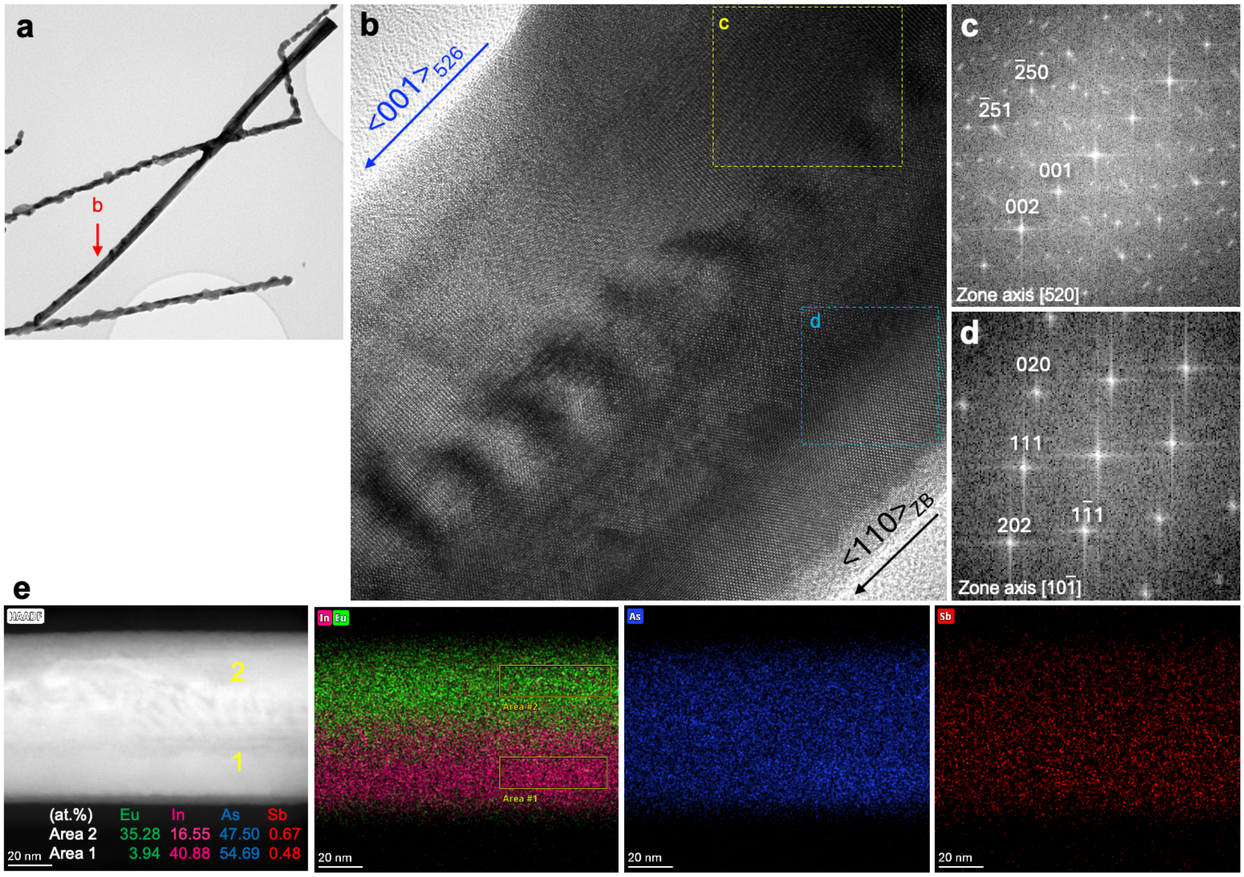

Figure S11 (a) TEM image of smoothly coated $Eu_5In_2As_6$ NWs, identical to Figure 3b in the main text. (b) Enlarged TEM image near the tip indicated by the red arrow in (a), showing that the <001> direction of $Eu_5In_2As_6$ is parallel to the <110> direction of ZB InAsSb. (c) FFT pattern from the area indicated by the yellow rectangle in (b), revealing the [520] zone axis of $Eu_5In_2As_6$. (d) FFT pattern from the area indicated by the blue rectangle in (b), showing the [10-1] zone axis of ZB InAsSb (e) HAADF image and corresponding EDS elemental maps of Eu, In, As, and Sb from (b). The inset in HAADF image provides EDS spectra quantification for Area 1 (InAsSb) and Area 2 ($Eu_5In_2As_6$).

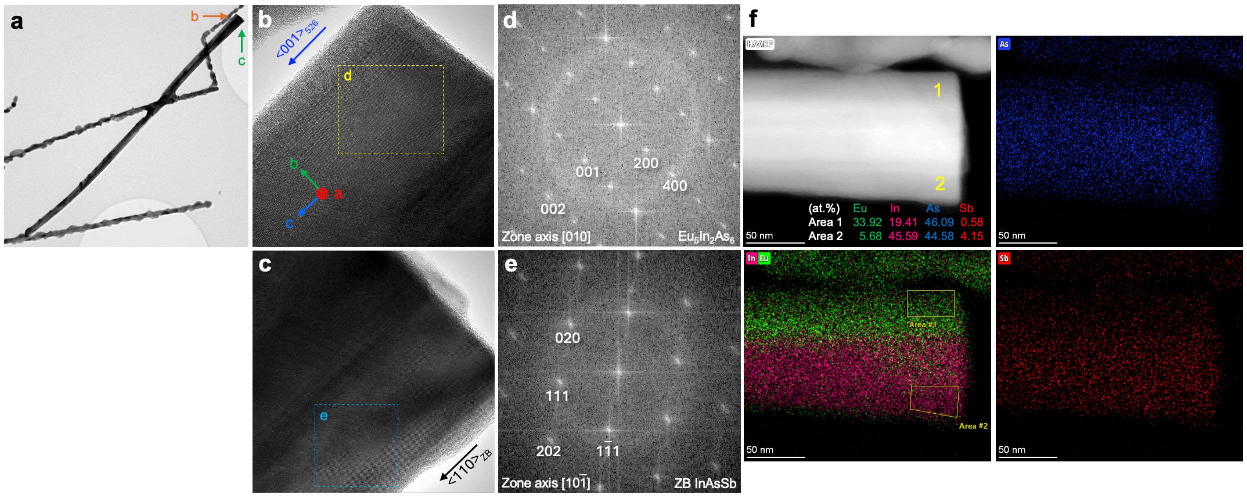

Figure S12 (a) TEM image of $Eu_5In_2As_6$ NWs with a smooth coating, identical to Figure S10a. (b, c) Enlarged TEM images near the bottom region indicated by the red and green arrows in (a), respectively, showing that the <001> direction of $Eu_5In_2As_6$ is parallel to the <110> direction of ZB InAsSb. (d, e) FFT patterns from the areas indicated by the yellow and blue rectangles in (b) and (c) reveal the [010] zone axis of $Eu_5In_2As_6$ and the [10-1] zone axis of ZB InAsSb, respectively. (f) HAADF image and corresponding EDS elemental maps of As, In, Eu, and Sb from (b, c). The inset in the HAADF image provides EDS spectra quantification for Area 1 (InAsSb) and Area 2 ($Eu_5In_2As_6$).

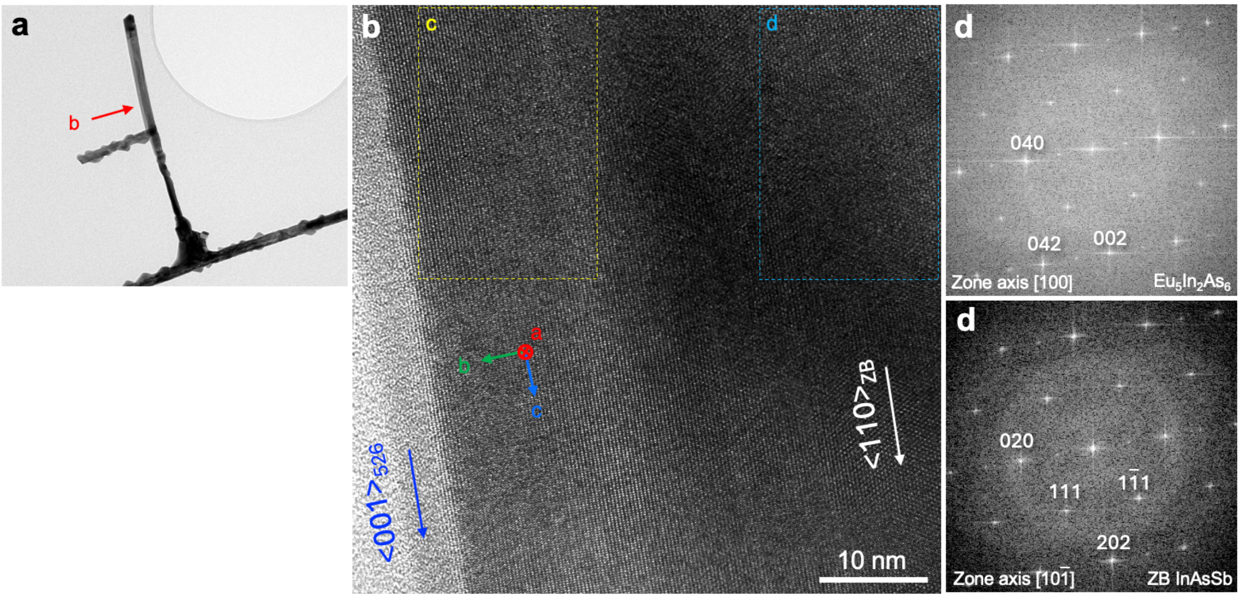

Figure S13 (a) TEM image of smoothly coated $Eu_5In_2As_6$ NWs. (b) Enlarged HRTEM image from the area indicated by the red arrow in (a), showing that the <001> direction of $Eu_5In_2As_6$ is parallel to the <110> direction of ZB InAsSb. (d, e) FFT patterns from the areas indicated by the yellow and blue rectangles in (b) reveal the [100] zone axis of $Eu_5In_2As_6$ and the [10-1] zone axis of ZB InAsSb, respectively.

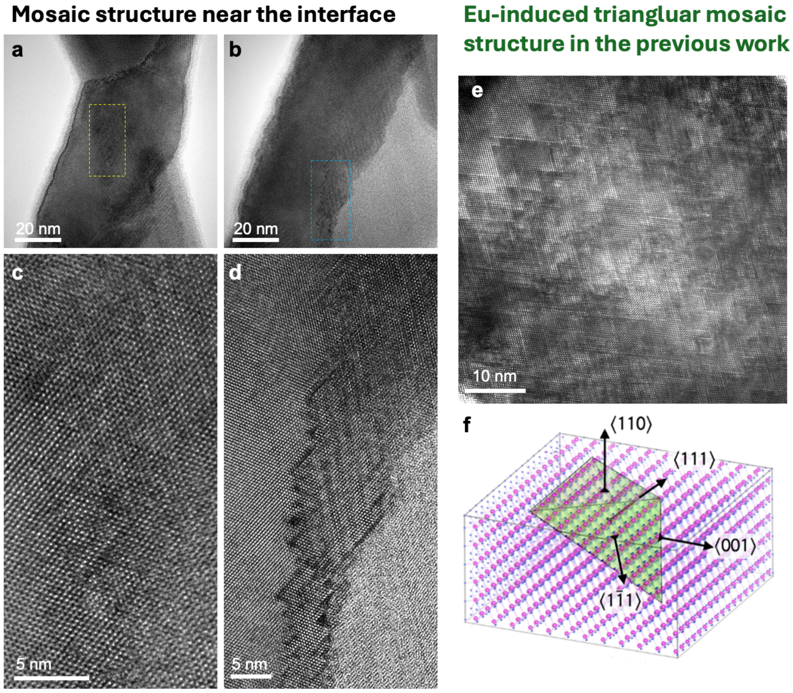

Figure S14. (a, b) TEM images of interface between the core NW and the Zintl grain along the <110> direction of ZB structure. (c, d) Enlarged TEM images of the areas indicated by the yellow and blue rectangles in (a) and (b), respectively, reveal a mosaic structure near the interface. (e) TEM image of the inversion domain boundary (IDB) network in the (EuIn)As NW[4]. (f) The 3D structure of the IDB is prismatic, bound by {111} planes[4]. The so-called mosaic structure in the ZB matrix results from the projection of a three-dimensional Eu atomic distribution.

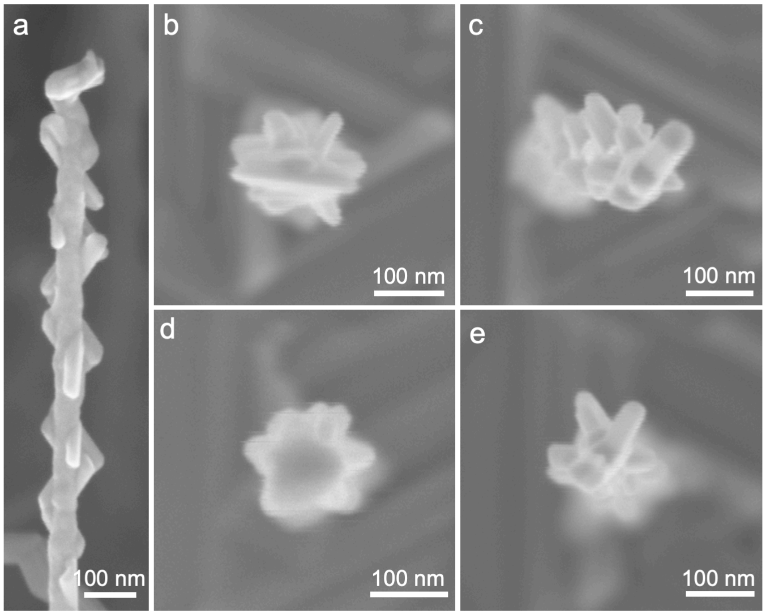

Figure S15 Three-fold symmetry of $Eu_5In_2As_6$ grains. (a) Side-view SEM image of an $Eu_5In_2As_6$ NW. (b–e) Top-view SEM images of the vertical $Eu_5In_2As_6$ NW.

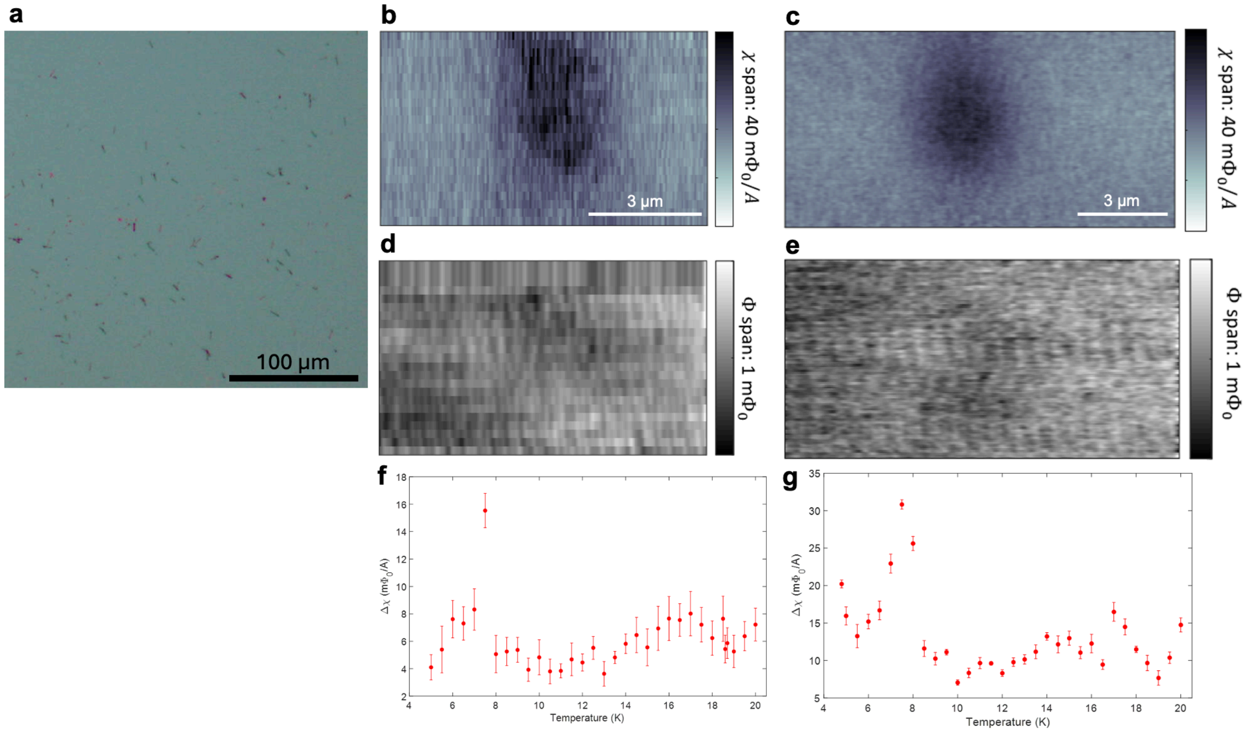

Figure S16 (a) Optical microscopy image of $Eu_5In_2As_5$ NWs on a Si/SiO_2 substrate. (b, c) AC susceptibility maps of the region in (a), taken at 5 K. (d, e) DC magnetism maps corresponding to the same regions as (b) and (c), respectively. (f, g) Plots of AC susceptibility versus temperature for the NWs measured in (b) and (c), respectively. The error bars represent the standard deviation of pixel values in the relevant areas in the vicinity of the maximum local signal.

Reference:

(1) Kang, J.-H.; Cohen, Y.; Ronen, Y.; Heiblum, M.; Buczko, R.; Kacman, P.; Popovitz-Biro, R.; Shtrikman, H. Crystal Structure and Transport in Merged InAs Nanowires MBE Grown on (001) InAs. *Nano Lett.* **2013**, *13* (11), 5190–5196. https://doi.org/10.1021/nl402571s.

(2) Kang, J.-H.; Krizek, F.; Zaluska-Kotur, M.; Krogstrup, P.; Kacman, P.; Beidenkopf, H.; Shtrikman, H. Au-Assisted Substrate-Faceting for Inclined Nanowire Growth. *Nano Lett.* **2018**, *18* (7), 4115–4122. https://doi.org/10.1021/acs.nanolett.8b00853.

(3) Kang, J.-H.; Galicka, M.; Kacman, P.; Shtrikman, H. Wurtzite/Zinc-Blende 'K'-Shape InAs Nanowires with Embedded Two-Dimensional Wurtzite Plates. *Nano Lett.* **2017**, *17* (1), 531–537. https://doi.org/10.1021/acs.nanolett.6b04598.

(4) Shtrikman, H.; Song, M. S.; Załuska-Kotur, M. A.; Buczko, R.; Wang, X.; Kalisky, B.; Kacman, P.; Houben, L.; Beidenkopf, H. Intrinsic Magnetic (EuIn)As Nanowire Shells with a Unique Crystal Structure. *Nano Lett.* **2022**, *22* (22), 8925–8931. https://doi.org/10.1021/acs.nanolett.2c03012.

(5) Clarke, J.; I. Braginski, A. *The SQUID Handbook: Fundamentals and Technology of SQUIDs and SQUID Systems, I*; John Wiley & Sons, Ltd, 2004.

(6) Persky, E.; Sochnikov, I.; Kalisky, B. Studying Quantum Materials with Scanning SQUID Microscopy. *Annual Review of Condensed Matter Physics* **2022**, *13* (1), 385–405. https://doi.org/10.1146/annurev-conmatphys-031620-104226.

(7) Song, M. S.; Houben, L.; Zhao, Y.; Bae, H.; Rothem, N.; Gupta, A.; Yan, B.; Kalisky, B.; Zaluska-Kotur, M.; Kacman, P.; Shtrikman, H.; Beidenkopf, H. Topotaxial Mutual-Exchange Growth of Magnetic Zintl Eu3In2As4 Nanowires with Axion Insulator Classification. *Nat. Nanotechnol.* **2024**, *19* (12), 1796–1803. https://doi.org/10.1038/s41565-024-01762-7.
